# Sensitive detection of colorectal cancer in peripheral blood by a novel methylation assay

**DOI:** 10.1186/s13148-021-01076-8

**Published:** 2021-04-23

**Authors:** Yunfeng Zhang, Qian Wu, Linhao Xu, Hong Wang, Xin Liu, Sihui Li, Tianliang Hu, Yanying Liu, Quanzhou Peng, Zhiwei Chen, Xianrui Wu, Jian-Bing Fan

**Affiliations:** 1 Department of Colorectal Surgery, The Sixth Affiliated Hospital, Sun Yat-Sen University, Guangzhou, 510655 Guangdong China; 2 Guangdong Provincial Key Laboratory of Colorectal and Pelvic Floor Diseases, The Sixth Affiliated Hospital, Sun Yat-Sen University, Guangzhou, 510655 Guangdong China; 3 AnchorDx Medical Co., Ltd, Unit 502, 3rd Luoxuan Road, International Bio-Island, Guangzhou, 510300 China; 4 AnchorDx Inc., 6305 Landing Pkwy, Fremont, CA 94538 USA; 5 Department of Pathology, School of Basic Medical Science, Southern Medical University, Guangzhou, 510515 China

**Keywords:** Liquid biopsy, Methylation biomarker, Colorectal cancer

## Abstract

**Background:**

Colorectal cancer (CRC) is a leading cause of cancer-related deaths worldwide. Early detection of CRC can significantly reduce its mortality rate. Current method of CRC diagnosis relies on the invasive endoscopy. Non-invasive assays including fecal occult blood testing (FOBT) and fecal immunological test (FIT) are compromised by low sensitivity and specificity, especially at early stages. Thus, a non-invasive and accurate approach for CRC screening would be highly desirable.

**Results:**

A new qPCR-based assay combining the simultaneous detection of the DNA methylation status of ten candidate genes was used to examine plasma samples from 56 normal controls, 6 hyperplastic polys, 9 non-advanced adenomas (NAAs), 22 advanced adenomas (AAs) and 175 CRC patients, using 10 ng of cfDNA. We further built a logistic regression model for CRC diagnosis. We tested ten candidate methylation markers including twist1, vav3-as1, fbn1, c9orf50, sfmbt2, kcnq5, fam72c, itga4, kcnj12 and znf132. All markers showed moderate diagnostic performance with AUCs ranging from 0.726 to 0.815. Moreover, a 4-marker model, comprised of two previously reported markers (c9orf50 and twist1) and two novel ones (kcnj12 and znf132), demonstrated high performance for detecting colorectal cancer in an independent validation set (*N* = 69) with an overall AUC of 0.911 [95% confidence interval (CI) 0.834–0.988], sensitivity of 0.800 [95% CI 0.667–0.933] and specificity of 0.971 [95% CI 0.914–1.000]. The stage-stratified sensitivity of the model was 0.455 [95% CI 0.227–0.682], 0.667 [95% CI 0.289–1.000], 0.800 [95% CI 0.449–1.000], 0.800 [95% CI 0.449–1.000] and 0.842 [95% CI 0.678–1.000] for advanced adenoma and CRC stage I-IV, respectively.

**Conclusion:**

kcnj12 and znf132 are two novel methylation biomarkers for CRC diagnosis. The 4-marker methylation model provides a new non-invasive choice for CRC screening and interception.

**Supplementary Information:**

The online version contains supplementary material available at 10.1186/s13148-021-01076-8.

## Introduction

Colorectal cancer (CRC) is among the three most common cancer types and the second leading cause of cancer related deaths worldwide [1]. Late-stage diagnosis is a major cause of morbidity and mortality of CRC. It is widely accepted that the disease burden can be decreased with proper population-based screening methods, which can detect precancerous lesions and early stage cancer [2]. Currently, CRC screening guidelines recommend colonoscopy and fecal immunochemical test (FIT) as the first-tier options [3]. However, due to the invasiveness, dietary restriction requirement and troublesome bowel preparation, colonoscopy has a low compliance rate [4]. The FOBT/FIT is a non-invasive test which is easy to perform and inexpensive, but it does not have sufficient sensitivity for advanced adenomas (AAs) or stage I CRC detection [4]. Another stool DNA-based colorectal screening test Cologuard was approved by the Food and Drug Administration (FDA) as a colorectal (CRC) screening method for average-risk adults [5]. Furthermore, based on a recent study evaluating the sample preference among screening-aged individuals, 78% of the survey participants preferred to choose blood test instead of the stool-based FIT [6]. Hence, a non-invasive and accurate blood-based test for early detection of CRC and adenomas could potentially improve patient compliance and is highly desirable.

Aberrant regulation of gene expression by DNA methylation is a well-characterized event in tumorigenesis and is commonly found in tumor suppressor genes among many cancer types, including CRC [7]. Increased levels of circulating-free methylated DNA in the blood of cancer patients have been reported [8]. Indeed, several promising blood-based epigenetic markers with high sensitivity and specificity have been identified in previous studies for the early diagnosis of CRC, but few of them have been validated extensively and commercially available [9–11]. Among them, *Epi proColon* (blood-based test that examines *sept9* methylation status) is the only blood-based test approved by FDA for CRC screening and has been used clinically for nearly 10 years [12]. However, the value of *sept9* promoter methylation as a CRC screening biomarker has been questioned due to its limited sensitivity, especially for early-stage cancers [13–15]. Owing to the suboptimal performance of commercially available epigenetic tests, discovery and validation of better candidate biomarkers or panels, which are robust in each of the stages of colorectal carcinogenesis from non-advanced adenomas (NAAs) to AAs and then to stages I-IV of CRC, is warranted.

One trend in cancer diagnosis to improve sensitivity is through combination of multiple markers and/or platforms, i.e. multi-omics. For example, it was reported that the sensitivities for CRC detection were 0.718 and 0.505, for *sept9* methylation and the carcinoembryonic antigen (CEA), respectively. When test results for *sept9* methylation and CEA were combined, the CRC detection rate was increased to 85.7% [16]. However, the limited amount of blood obtained during a routine test could curb the assay numbers and the potential of maximizing the diagnostic performance, esp. for cell-free DNA (cfDNA) based markers.

Our recent work identified a novel CRC-specific methylation model at tissue level, which was highly sensitive to early-stage CRC and stage-dependent [17]. This model consisted of markers that have been reported previously, e.g. *itga4*, *thbd*, *vav3-as1* and a few novel ones, including *kcnj12*, *znf132*, and *sfmbt2*. In this study, we evaluated the performance of these methylation markers in the plasma cohort, and further refined the methylation model for the application in CRC early detection.

## Results

### Patients and samples

**Fig. 1:**
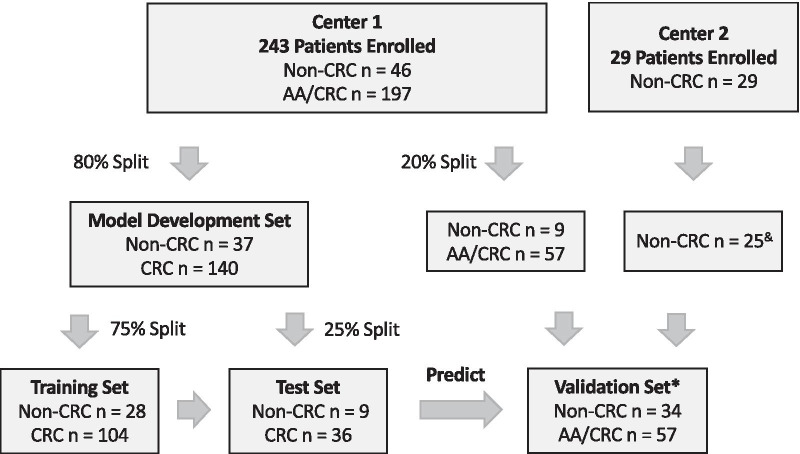
The study design of development and validation of a cfDNA-based methylation assay for colorectal cancer diagnosis. * advanced precursor cancer lesions only in validation dataset ^&^ 4 samples excluded due to hemolysis or or inadequate cfDNA amount extracted from plasmas

A total of 272 patients and controls, including 175 CRC patients, 22 patients with advanced adenomas (AAs), 9 patients with non-advanced adenomas (NAAs), 6 patients with hyperplastic polys and 60 healthy controls (Fig. 1) were enrolled in this study from two clinic centers; their plasma samples were used for model development and validation. Of which, 4 samples were excluded from analysis due to hemolysis or inadequate cfDNA amount extracted from plasmas. Relevant clinical characteristics of the cohort including age, gender, histology and tumor staging (AJCC criteria) were listed in Table 1. Among the 268 patients and controls, the percentage of male patients was 46.7% (53.3%, female). The overall median age was 58 years. There were no significant differences of age and gender between non-CRC and CRC groups. Stage I + II cancers comprised 40.0% of the total CRC patients.

**Table 1:** The demographic and clinical characteristics of the study cohort

	Non-CRC			AA/CRC		
	Normal	Hyperplastic polys	Non-advanced adenomas	Advanced adenoma	Tumor	Total
Total (*n*)	56	6	9	22	175	268
Age (years)	46 (27–70)	50 (38–51)	53 (38–76)	57.5 (34–86)	60 (24–89)	58 (24–89)
Gender—no. (%)						
Male	27	2	2	13	81	125 (46.7%)
Female	29	4	7	9	94	143 (53.3%)
Stage						
Stage I					26 (14.8%)	
Stage II					44 (25.2%)	
Stage III					21 (12.0%)	
Stage IV					84 (48.0%)	

### Methylation status of different biomarkers in plasma samples

**Fig. 2:**
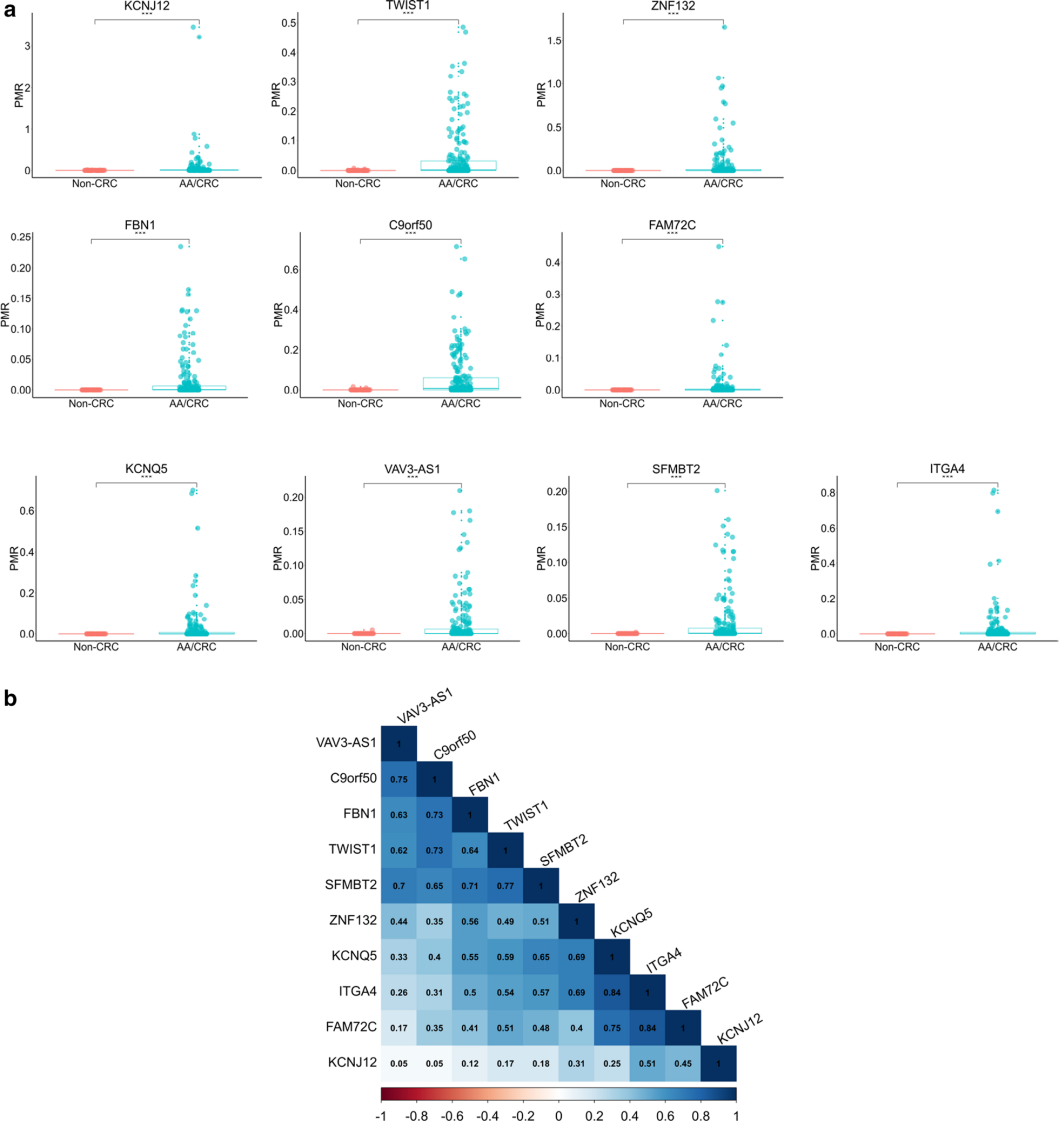
DNA methylation of 10 candidate biomarkers. **a** DNA methylation levels, measured by PRM, of ten markers TWIST1, VAV3-AS1, FBN1, C9orf50, SFMBT2, KCNQ5, FAM72C, ITGA4, KCNJ12 and ZNF132 in 71 non-CRC samples and 197 CRC samples. * *p* < 0.05; ** *p* < 0.01; *** *p* < 0.001. **b** Correlation of DNA methylation levels in CRC plasma samples of the 10 markers. Blue, positively correlated; Red, negatively correlated

The methylation status of 10 candidate markers (*twist1*, *vav3-as1*, *fbn1*, *c9orf50*, *sfmbt2*, *kcnq5*, *fam72c*, *itga4*, *kcnj12*, *znf132*) were analyzed in all plasma samples (*N* = 268) using a qPCR-based assay (Additional file 1: Figure S1 and Additional file 1: Table S1), divided into non-CRC group (Normal, HP, NAA) and AA/CRC group (AA and stage I-IV CRC). A percentage of methylated reference (PMR) score was used to measure the methylation levels. After evaluating the methylation level of each marker in AA/CRC group and non-CRC group, we found all 10 targeted genes showed significantly higher PMR values in AA/CRC group compared to non-CRC group, indicating the higher frequency of methylation in tumors (Fig. 2a). The performance characteristics of these markers were shown in Table 2. The area under curves (AUCs) of these markers ranged from 0.726 to 0.815, with *igta4* achieved the highest AUC value of 0.815, *fbn1* and *kcnq5* showed a mean specificity of 100% in 2,000 bootstrap samplings. Even though the AUCs of these markers in plasma were lower than what was shown in the tissue [17], all 10 markers had the diagnostic potential for detecting CRC in blood. We also found PMRs of these 10 markers showed a trend of stepwise increase with the progression of CRC (Additional file 1: Figure S2), indicating these markers maybe useful for cancer staging. Furthermore, the analysis of DNA methylation levels of these ten markers suggested that there were strong correlations between *kcnq5* and *itga4*, *fam72c* and *itga4* (coefficient > 0.800, Fig. 2b).

**Table 2:** The single biomarker performance metrics

Marker	AUC	Sensitivity	Specificity
twist1	0.765 (0.716–0.815)	0.563 (0.497–0.645)	0.972 (0.901–1.000)
vav3-as1	0.743 (0.705–0.782)	0.503 (0.431–0.574)	0.986 (0.944–1.000)
fbn1	0.786 (0.747–0.826)	0.584 (0.508–0.66)	1.000 (0.958–1.000)
c9orf50	0.771 (0.728–0.814)	0.594 (0.523–0.66)	0.972 (0.930–1.000)
sfmbt2	0.781 (0.744–0.818)	0.574 (0.503–0.64)	0.986 (0.958–1.000)
kcnq5	0.726 (0.689–0.763)	0.467 (0.396–0.533)	1.000 (1.000–1.000)
fam72c	0.785 (0.746–0.823)	0.584 (0.503–0.66)	0.972 (0.915–1.000)
itga4	0.815 (0.779–0.851)	0.64 (0.569–0.711)	0.986 (0.944–1.000)
kcnj12	0.799 (0.745–0.853)	0.624 (0.457–0.878)	0.887 (0.606–0.986)
znf132	0.764 (0.718–0.81)	0.563 (0.487–0.635)	0.972 (0.915–1.000)

### Development and validation of the diagnostic model for CRC early detection

All the plasma samples were divided randomly into two sets with a 4:1 ratio: a model development set (normal *N* = 24; non-advanced-precancerous lesions *N* = 13; CRC *N* = 140) and a validation set (normal *N* = 32; non-advanced-precancerous lesions *N* = 2; advanced-precancerous lesions *N* = 22; CRC *N* = 35). The model development set was comprised of a training set (normal *N* = 17; non-advanced-precancerous lesions *N* = 11; CRC *N* = 104) and a test set (normal *N* = 7; non-advanced-precancerous lesions *N* = 2; CRC *N* = 36) in a way that the age and gender in the testing set is matched to the training set. Due to limited samples, all high-grade intraepithelial adenocarcinoma (HGIN) samples, i.e. advanced adenomas, were grouped into the validation set (Additional file 1: Table S2).

**Fig. 3:**
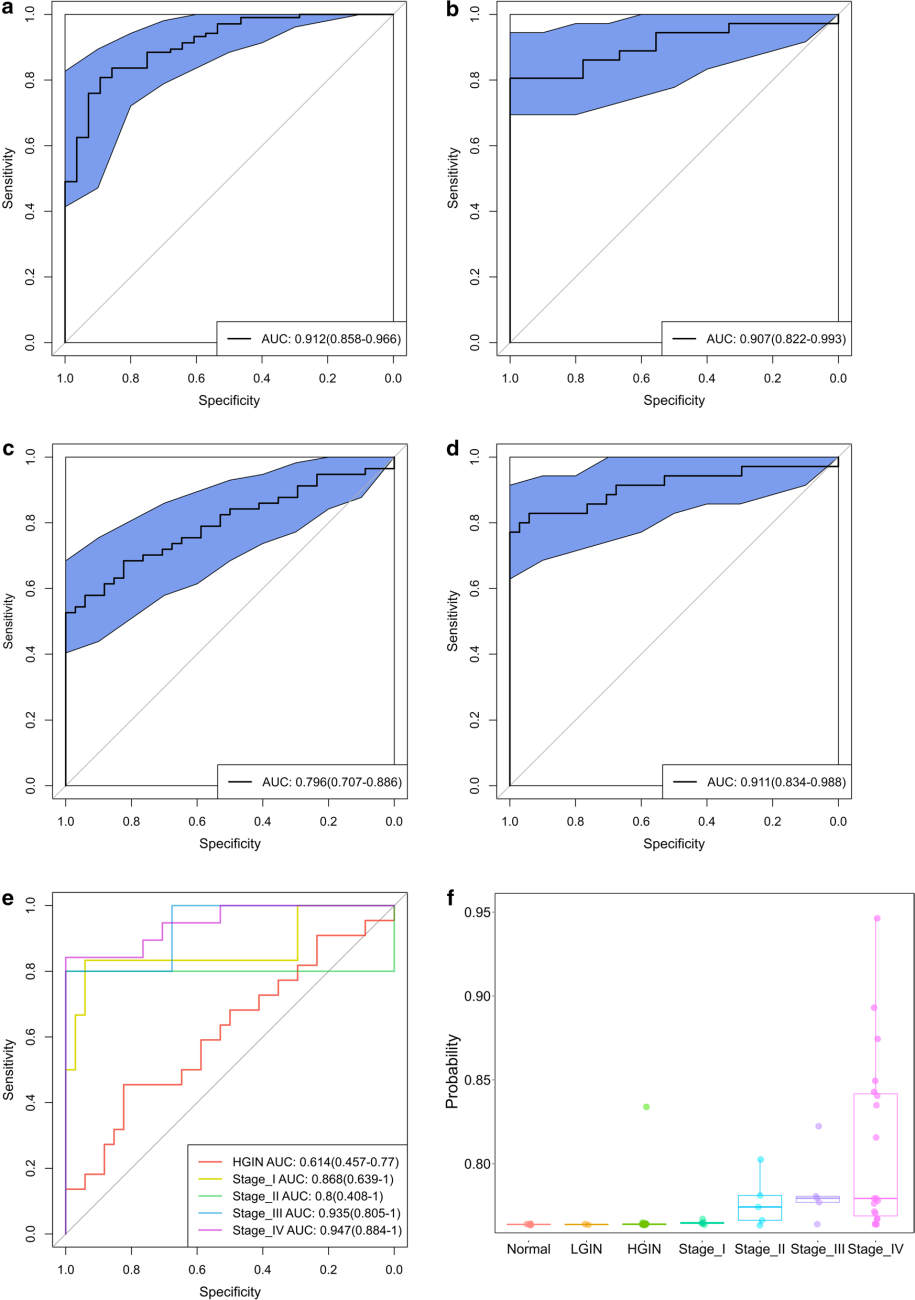
4-marker model performance in CRC diagnosis. A representative receiver operating curve (ROC) displays the classification performance of the 4-marker model. **a** In the training set, the area under the curve (AUC) was 0.912. **b** In the test set, the AUC was 0.907. **c** In the validation set with advanced adenomas, the AUC was 0.796. **d** In the validation set without advanced adenomas, the AUC was 0.911. **e** The performance of the 4-marker model in detecting different stage CRC. HGIN, AUC 0.614. Stage I CRC, AUC 0.868. Stage II CRC, AUC 0.800. Stage III CRC, AUC 0.935. Stage IV CRC, AUC 0.947. **f** The risk score of the 4-marker model in detecting different stage CRC

We first used all 10 markers to identify the best performing marker combination in 100 splits of the data set using logistic regression, which achieved AUCs of 0.916 [0.863–0.969] and 0.910 [0.826–0.995] in the training and test sets, respectively (Additional file 1: Figure S3A, B). *c9orf50* performance ranked the highest in the 10-marker model regarding the feature coefficient (Additional file 1: Figure S3C). A recursive feature elimination process was used to search for all marker combinations that could maintain an AUC within 1% of the 10-marker model’s test AUC (0.910) using the smallest number of markers. This resulted in a combination of four genes, *c9orf50*, *kcnj12*, *znf132* and *twist1*. As shown in Fig. 3a, b, the AUC in the test set was 0.907 [0.822–0.993], significantly higher than the individual gene models. All four markers in the model had very similar coefficients, indicating the four markers contributing more of less equally to the model performance (Additional file 1: Table S3). The 4-marker model had an overall sensitivity of 0.806 [0.676–0.935] and specificity of 0.889 [0.684–1.000] in the test set, compared to individual markers’ sensitivities of 0.594, 0.640, 0.559 and 0.579 and specificities of 0.957, 0.870, 0.957 and 0.957 for *c9orf50*, *kcnj12*, *znf132* and *twist1*, respectively (Table 3). To further validate whether the performances of these four markers could be extrapolated to other ethnicities, we calculated the performance of each individual marker on two CRC groups, colon adenocarcinoma (COAD) and rectum adenocarcinoma (READ), comprised mostly of White/Black from TCGA database. As expected, each marker achieved a superior performance in diagnosing CRC with an AUC ranging from 0.642 to 0.962 in cohort COAD and 0.661 to 0.977 in cohort READ, as shown in Additional file 1: Figure S4.

**Table 3:** The 4-marker model performance metrics

		Sensitivity		Specificity		Sensitivity		
	Overall	All_rmHGIN*	Overall	HGIN	Stage_I	Stage_II	Stage_III	Stage_IV
Train	0.808 (0.732–0.883)	0.808 (0.732–0.883)	0.893 (0.778–1.000)	–	0.786 (0.571–1.000)	0.706 (0.553–0.859)	0.818 (0.59–1.000)	0.889 (0.797–0.981
Test	0.806 (0.676–0.935)	0.806 (0.676–0.935)	0.889 (0.684–1.000)	–	0.833 (0.535–1.000)	0.600 (0.171–1.000)	0.800 (0.449–1.000)	0.85 (0.694–1.000)
Validation	0.544 (0.415–0.673)	0.800 (0.667–0.933)	0.971 (0.914–1.000)	0.455 (0.227–0.682)	0.667 (0.289–1.000)	0.800 (0.449–1.000)	0.800 (0.449–1.000)	0.842 (0.678–1.000)

*Removed HGIN (AA) samples

We then tested this 4-marker model in an independent validation set. The overall AUC dropped significantly to 0.796 [0.707–0.886] (Fig. 3c). However, if ruled out the AA samples in the validation set, the AUC increased to 0.911 [0.834–0.988] and was consistent with the diagnostic performance in the test set (Fig. 3d). The sensitivity of the model in AA group was 0.455 [0.227–0.682]. The sensitivity and specificity of the model in the validation set without AA was 0.800 [0.667–0.933] and 0.971 [0.914–1.000], respectively (Table 3). These data suggested that the 4-marker model had an enhanced detection sensitivity in detecting all stages CRC compared to individual markers.

### Model performance in different stages with CRC progression

We then evaluated the performance of this model in different stages of CRC. The 4-marker model showed a limited diagnostic performance in detecting AA, with an AUC of 0.614 [0.457–0.770], while achieved an AUC of 0.868 [0.639–1.000], 0.800 [0.408–1.000], 0.935 [0.805–1.000], 0.947 [0.884–1.000] for stage I, II, III and IV, respectively (Fig. 3e). The sensitivity was 0.667 [0.289–1.000], 0.800 [0.449–1.000], 0.800 [0.449–1.000] and 0.842 [0.678–1.000] for stage I-IV, respectively (Table 3). The distributions of CRC risk probability generated from the model in different stage groups were shown in Fig. 3f and demonstrated a stepwise increasing trend.

## Discussion

CRC is a leading cause of cancer-related deaths worldwide. Patient non-compliance and the compromised performance of FIT-based screening strategies limit their utility in current CRC screening market. The strategy of an easily administered blood-based screening test for the early detection of CRC followed by colonoscopy validation has the potential to become an effective practice for reducing the mortality of this disease. Here, we reported the development of a novel blood-based model using four CRC-specific DNA methylation markers, *c9orf50*, *kcnj12*, *znf132* and *twist1*, and demonstrated its potential for the detection of CRC in routine physical examinations.

At present, *sept9* methylation test, provided by a commercial name *Epi proColon*, was the only blood-based epigenetic test approved by FDA and National Medical Products Administration (NMPA, China) for CRC screening. However, its sensitivity is rather limited: one clinical trial reported that the sensitivities of *Epi proColon* for detecting stages I–IV CRC were 0.350, 0.630, 0.460, and 0.774, respectively [13]. Another large cohort study of 1,544 plasma samples showed the test had a sensitivity of 0.682 and a specificity of 0.782 [15]. Another widely used biomarker CEA also showed a limited sensitivity ranging from 0.080 to 0.466 depending on the CRC stages [18]. Even though studies have shown a combination of methylated *sept9* methylation with CEA could significantly increase the sensitivity of CRC detection, the specificity was decreased significantly [16]. Therefore, the potential best test should be the one that best balances sensitivity and specificity.

In this study, we developed a new blood-based early CRC screening test, which combined two previously reported methylation markers, *c9orf50* and *twist1*, and two novel genes, *kcnj12* and *znf132*. Methylated *twist1* and *c9orf50* have been studied as novel blood-based markers for CRC detection recently [17, 19, 20]. Their diagnostic performances have been validated in both fecal and serum/plasma samples. *kcnj12* belongs to the inward-rectifier potassium channel family, which plays an important role in regulating K^+^ channels and one study suggests that it may induce the growth of cancer cells by regulating RelA-activated NF-κB signaling [21]. The overexpression of ion channels and NF-κB signaling have been reported in multiple cancers, including colon cancer [22, 23]. However, there is limited reports demonstrating *kcnj12* mutation or aberrant expression in colon cancer. As for *znf132*, methylation mediated silencing events have been validated in esophageal squamous cell carcinoma and breast cancer, however, not in colon cancer [24, 25].

Compared with methylated *sept9* methylation test, our model achieved a superior performance in detecting CRC with an overall sensitivity of 0.800 and specificity of 0.971, comparable to the FIT test with a sensitivity of 0.790 and specificity of 0.940. The model also had relative high sensitivities, 0.667 and 0.800, in stage I and II CRC, respectively. This suggests that the 4-marker panel has the potential as a screening test for CRC. The model could also be used to supplement existing FIT-based CRC screenings, e.g., as an option for the patients that refuse using the FIT test, or for triaging FIT-positives to colonoscopy to reduce the number of colonoscopies needed.

Our study has a few limitations. Firstly, the 4-marker model shows a limited diagnosis performance on the advanced adenoma plasma samples (sensitivity 0.455 and specificity 0.971). Even though the 4-marker model has a less optimized performance on advanced adenoma compared to other CRC stages, the overall sensitivity and specificity are still superior to or comparable with current established methods, e.g. blood-based mSEPT9 (sensitivity 0.171 and specificity 0.945) [26] and CEA (sensitivity 0.08 and specificity 0.700) [27], stool-based FOBT (sensitivity 0.122 and specificity 0.919) [26] and FIT (sensitivity 0.400 and specificity 0.940) [28], as well as a few published non-invasive CRC marker studies to date [29–34]. Theoretically, the diagnosis performance for advanced adenoma could further be improved by integrating other complementary markers with high sensitivity and low specificity, to develop into a multi-omics assay [32]. A larger study with more adenoma samples is needed to further examine and optimize the model by adding new markers to increase the sensitivity for detecting adenomas, which is critical to reduce CRC incidence. Secondly, we enrolled CRC patients with known cancers, most of whom were diagnosed on the basis of symptoms. Therefore, the late-stage tumor ratio within these patients would be higher than those asymptomatic individuals in a routine screening scenario. Consequently, the sensitivity of detection in a screening population might be less than that reported here. Thirdly, there is a possibility that the markers might also become positive in the plasma of some other cancer types. Therefore, future studies are needed to investigate the model’s specificity against other cancer types.

In summary, we have screened 10 candidate methylation markers in the blood and developed a sensitive and specific 4-marker model for CRC early diagnosis. Once validated in a larger and asymptomatic clinical setting, this model could provide a new non-invasive choice for early tumor detection and interception in CRC.

## Methods

### Study population

Total 243 patients and healthy individuals with informed consent were enrolled in Sixth Affiliated Hospital of Sun Yat-sen University from 2016 to 2019, and total 29 healthy individuals were enrolled in Shenzhen People’s Hospital in 2021.

Blood samples were collected in the hospital admission day or 1 to 8 days prior to surgery using EDTA Tubes (BD, Cat# 367525). Plasma was separated within 2 h after receiving the whole blood samples using a standard protocol and stored at -80 °C until use. The plasma volume ranged from 3.0 to 5.0 ml. Repeated freezing and thawing of plasma were avoided to prevent cfDNA degradation and genomic DNA contamination from white blood cells (WBC). The normal cohort included the hospitalized patients who had physical examination or suffered from haemorrhoids, anal fissure or constipation, but without any benign, precancerous or cancerous lesions confirmed by colonoscopy.

The human methylation 450 K array data set and clinical characteristics of colon adenocarcinoma (COAD) and rectum adenocarcinoma (READ) were obtained from The Cancer Genome Atlas (TCGA).

### cfDNA extraction, bisulfite treatment and methylation analysis

Cell-free DNA (cfDNA) was isolated by the MagMAX™ Cell-Free DNA Isolation Kit (Thermo Fisher Scientific, Cat# A29319) according to the manufacture’s protocol. The concentration of cfDNA was measured by Qubit™ dsDNA HS Assay Kit (Thermo Fisher Scientific, Cat# Q32854) and the quality was examined using the Agilent High Sensitivity DNA Kit (Cat# 5067–4626) on a Agilent 2100 Bioanalyzer Instrument. 10 ng of cfDNA without overly genomic DNA contamination were proceeded to perform the following assays.

Bisulfite conversion was performed using the Zymo EZ DNA Methylation-Direct Kit (Zymo Research, Cat# D5044) according to the manufacturer’s protocol. Briefly, cfDNA was first denatured at 98 °C for 10 min, then treated with sodium bisulfite at 64 °C for 3.5 h. Then, DNA was added into a spin column and desulphonated by adding desulphonation buffer and incubated at room temperature for 20 min. Bisulfite-converted DNA was purified by a spin column and eluted with 25 μL of Elution Buffer.

The bisulfite-modified DNA were further analyzed by a CRC 10-Gene Meth-Detect Kit according to the manufacturer’s instruction (AnchorDx, China, Cat# CRCB012). In short, we used a strategy of multiplexing PCRs followed by the quantitative methylation-specific PCR (qMSP) quantification for each candidate methylation marker to overcome the limited quantity of cfDNA in detecting ten methylation markers simultaneously (Additional file 1: Figure S1), as previously reported [35, 36]. A multiplex 10-marker targeted pre-amplification of methylated regions was performed on 15 μl of the bisulfite-treated DNA using 25 μl of the Meth-Pre-Amp Master Mix (AnchorDx, China, Cat# CRCB012-01) and 10 μl of Meth-Pre-Amp 10-Gene CRC Panel (AnchorDx, China, Cat# CRCB012-02, primer sequence shown in Additional file 1: Table S1) in the kit in a thermal cycler (Thermo Fisher, United States, Cat# 4484073) under the following cycling conditions: denaturation at 98 °C for 30 s, 5 cycles (98 °C for 15 s, 58 °C for 15 s and 72 °C for 15 s), 17 cycles (98 °C for 15 s, 63 °C for 15 s and 72 °C for 15 s), final extension at 72 °C for 5 min and hold at 4 °C.

The amplified products were diluted 2X before used as template for quantitative methylation-specific PCR on the QuantStudio 3 Real-Time PCR System (Thermo Fisher, United States) using Meth-Quant Master Mix (AnchorDx, China, Cat# CRCB012-03) and 10-Gene CRC Detect Panel (AnchorDx, China, Cat# CRCB012-04, primer/probe sequence shown in Additional file 1: Table S1) under the following cycling conditions: denaturation at 98 °C for 30 s, 40 cycles (95 °C for 10 s, 62 °C for 20 s), with fluorescent signal collected at the annealing/extension step (62 °C for 40 s).

### Modeling and statistical analysis

The percentage methylated reference (PMR) was calculated by dividing the dividing the normalized quantity (target gene/reference gene ratio) of the samples by the normalized quantity of the methylation positive controls and multiplying by 100 [37]. Briefly, a standard curve between ΔCt and methylation percentage was established for each marker using different methylation standards (0%, 0.1%, 0.33%, 1%, 3.3%, 10%, 33%, 66% and 100% in current study). PMR was calculated based on the function of ΔCt and methylation percentage obtained from the standard curve. ACTB was used as a default reference for normalization.

The logistic regression-based models were constructed using Python Sklearn packages. For a single gene marker, we used the target gene and the reference gene ACTB for fitting. The optimal threshold was determined by Youden's index. For the model development, we randomly divided the whole cohort into two parts, the model development set (80%) and the validation set (20%). The model development set was used for model building and testing while the validation set for evaluating the model performance. The performance of each model was evaluated by area under receiver operating characteristic curve (AUC) using the R package pROC.

We used Fisher exact statistic test for categorical variables including gender and Wilcoxon test for continuous variables like age. All statistical analysis was performed with R software, version 3.32. Unless otherwise specified, all statistical tests were two-sided.

## Supplementary Information

**Additional file 1**. Supplemental Figures and Tables.

## Data Availability

The datasets used and/or analyzed during the current study are available from the corresponding author on reasonable request.

## Abbreviations

AUC: Area under curve
CRC: Colorectal cancer
HP: Hyperplastic polys
NAA: Non-advanced adenomas
AA: Advanced adenomas
PMR: Percentage of methylated reference
