# Supplementary material for: Sensitive detection of colorectal cancer in peripheral blood by a novel methylation assay
Source: Clin Epigenetics. 2021 Apr 23;13:90. doi: 10.1186/s13148-021-01076-8 (PMC8066866; doi:10.1186/s13148-021-01076-8)
Supplement: Supplementary file 1 — Additional file 1. Supplemental Figures and Tables. [file 13148_2021_1076_MOESM1_ESM.pdf]

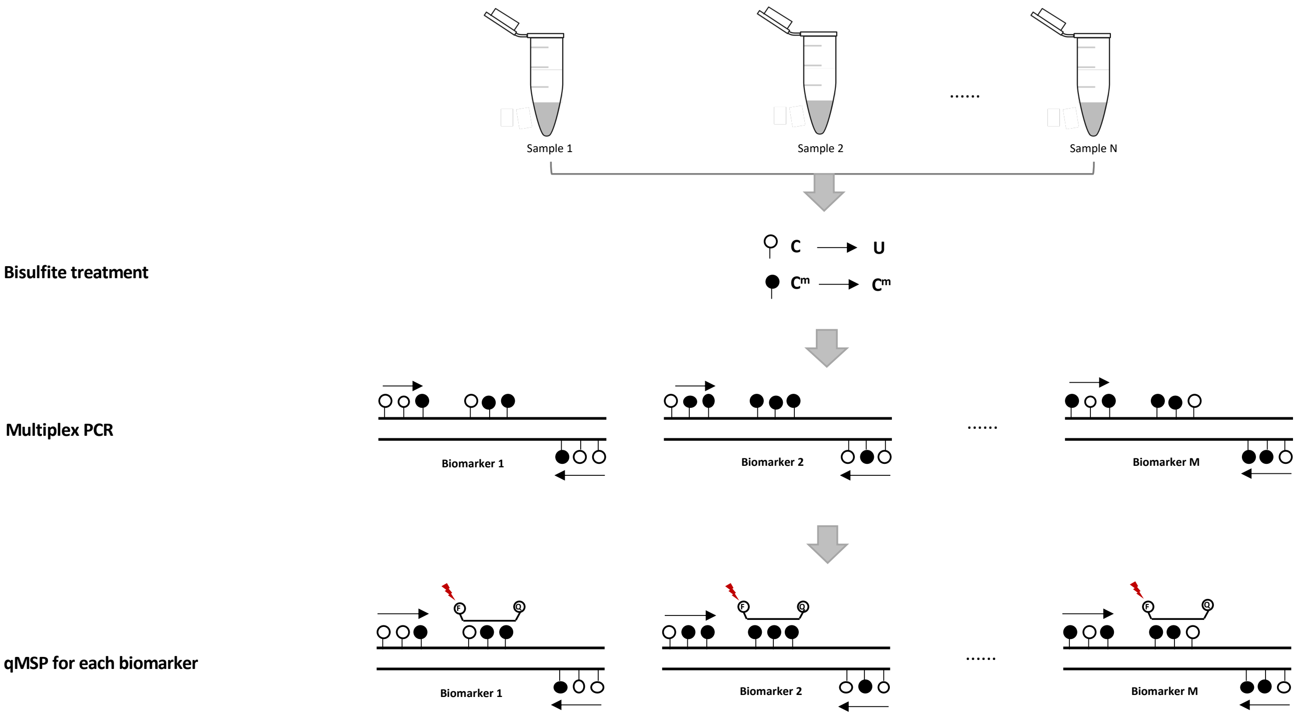

Figure S1 The strategy of using mPCRs followed by quantitative methylation-specific PCR (qPCR) quantification for each candidate marker used in the study.

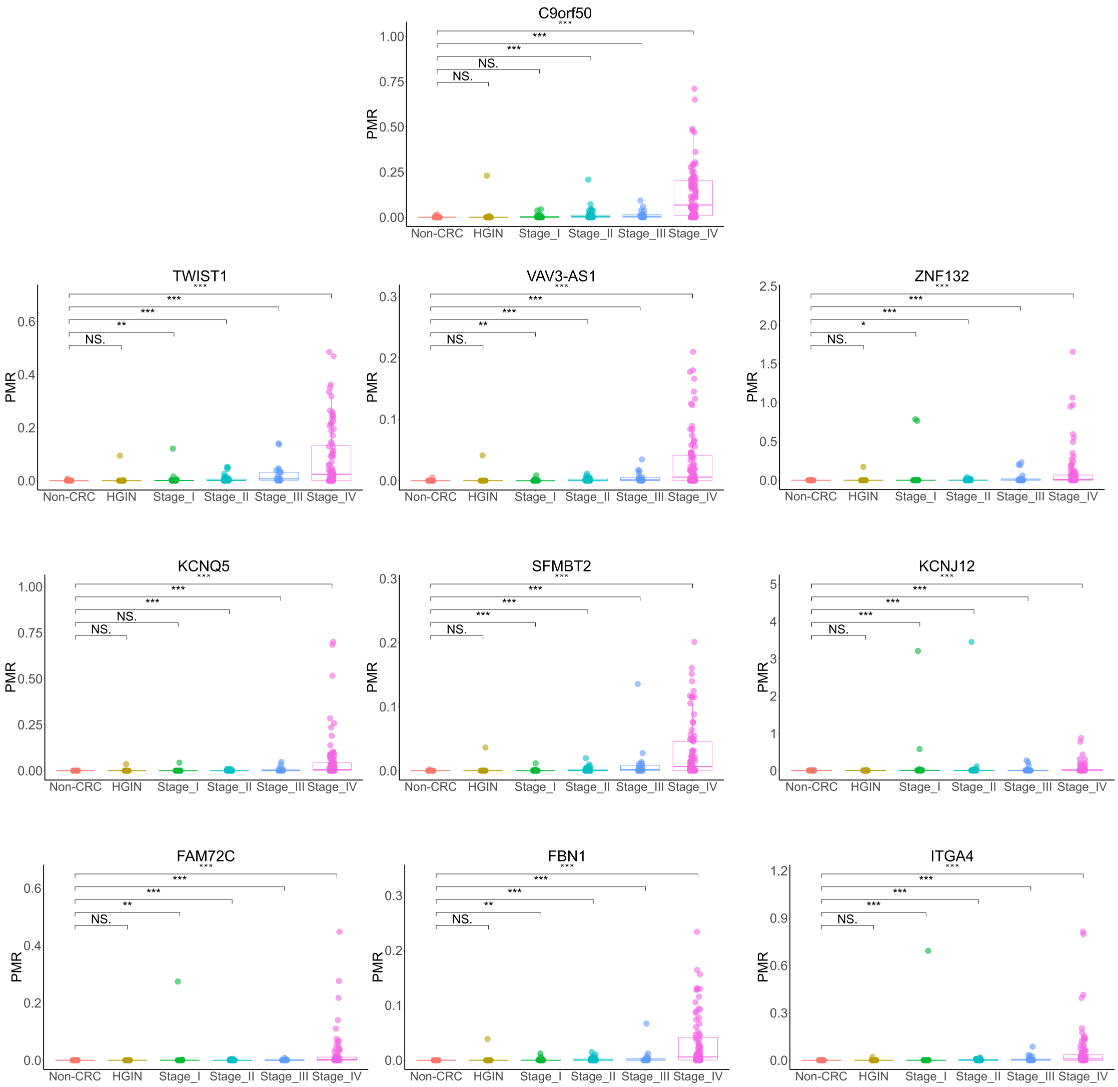

Figure S2 The PMR value of 10 markers in different stages of CRC. * p<0.05 ** p<0.01 *** p<0.001

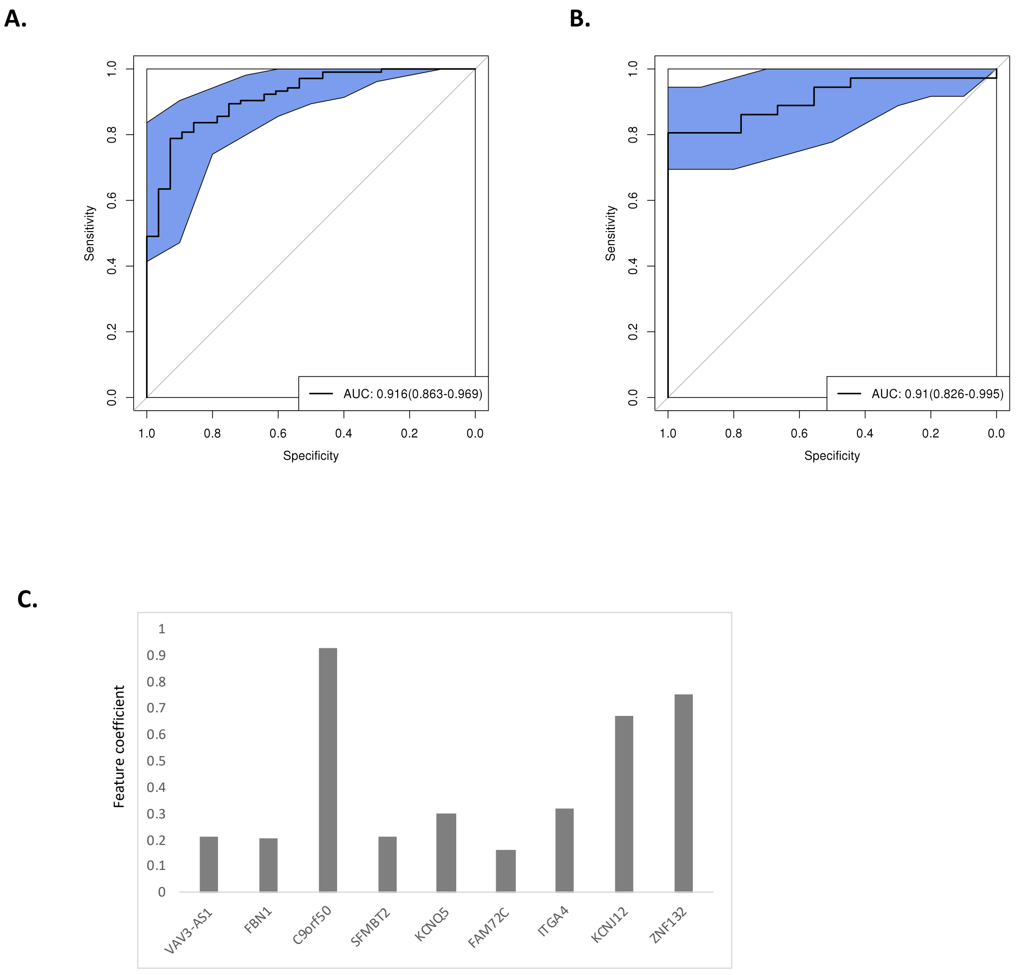

Figure S3 The diagnostic performance of 10-marker LR model. (A) In the training set, the AUC was 0.916 (B) In the test set, the AUC was 0.910 (C) The feature coefficient of the 10-marker model

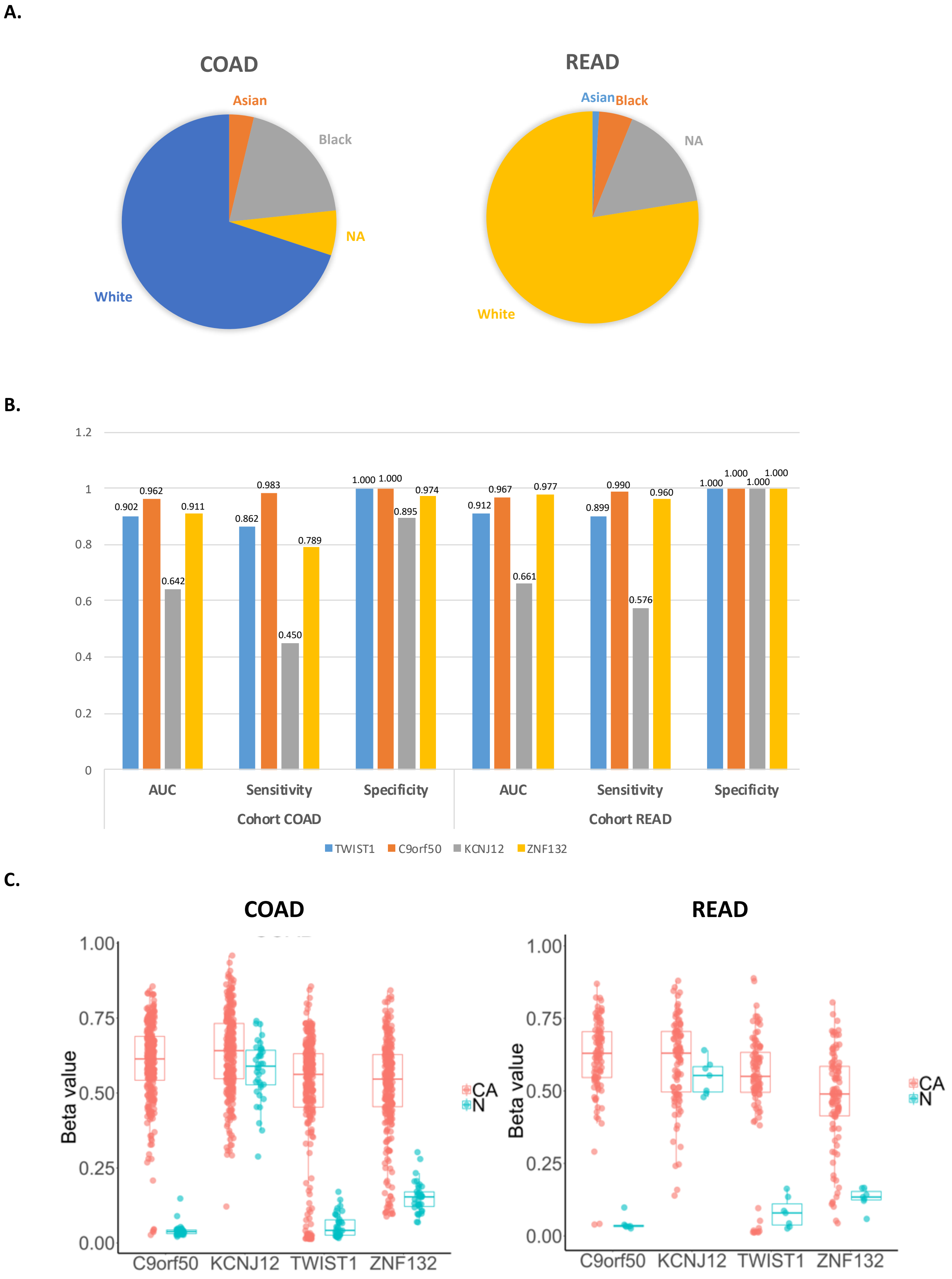

Figure S4 The diagnostic performance of 4 marker in colon adenocarcinoma (COAD) and rectum adenocarcinoma (READ) populations from TCGA. (A) Ethnic summary of TCGA cohort COAD (N=334) and cohort READ (N=105) (B) Performance of individual marker in TCGA cohort COAD (Normal N=38, Case N=296) and cohort READ (Normal N=7, Case N=98) (C) Methylation level distribution for each marker in case group (CA) and control group (N)

Table S1 Primers and probes used in the study

Target	Primer & Probe	Sequence5'_3'
ITGA4	ITGA4-F	TGCGGAGGCGTAGGGTC
	ITGA4-R	CAACCGAAATTCCCCAACG
	**ITGA4-P**	CCTACAACCGCGCGTAAACAAAAACG
TWIST1	TWIST1-F	GTAGCGCGGCGAACGT
	TWIST1-R	AAACGCAACGAATCATAACCAAC
	**TWIST1-P**	CCAACGCACCCAATCGCTAAACGA
FBN1	FBN1-F	GCGATTTTTAGTAGACGTTTTCGAC
	FBN1-R	AACCCCGAACGCGACA
	**FBN1-P**	ACGACGAAAACCGATAACGACTCGACATC
SFMBT2	SFMBT2b-F	AAAACGTTTTTAGGTATTTGGTCG
	SFMBT2b-R	ACGATCCCCGAAACTAACG
	**SFMBT2-P**	CTCCTCCGAACCCGCGAACCTAATC
C9orf50	C9orf50-F	GACGCGTCCCGAAAATC
	C9orf50-R	TTTTTTAGGAAGGCGTTTAAGAAGT
	**C9orf50-P**	AAAACGCGAACGCCCCCGA
ZNF132	ZNF132-F	GGAATGGCGTTTATTGCGTC
	ZNF132-R	CCCGAAAATACCTATCTCCTCGA
	**ZNF132-P**	CAACCCTAAAACACCGCGAAAATCCTTC
KCNQ5	KCNQ5-F	CTCTACAACCCCTCCTTTCCC
	KCNQ5-R	CGTTCGTTAGTTAGAGATTTTTGAGTC
	**KCNQ5-P**	CCCCGAAACCCGAACGATTTACTAACTACC
VAV3-AS1	VAV3-AS1-F	CCTTACGAAACTCACGCAACC
	VAV3-AS1-R	GGGTTTTGGGGGATTTTATCG
	**VAV3-AS1-P**	CCTAAAATCTTCTCTCGAAACGACTTCCCG
FAM72C	FAM72C-F	TTGGTCGGTTGAGTTTGGTTTC
	FAM72C-R	CACTTTCACTAAAACTAAAACAAAATTTACCG
	**FAM72C-P**	AACCTCCCGAAACCGACCAATAAAATCGAA
KCNJ12	KCNJ12-F	AAAAACAAAAACTACCTAAATTTACAACGC
	KCNJ12-R	GATTTGTTTGCGCGTTCGAG
	**KCNJ12-P**	AACTTCCCTAAAACTCGACGACCGCCCTA
ACTB	ACTB-F	GTGATGGAGGAGGTTTAGTAAGTT
	ACTB-R	CCAATAAAACCTACTCCTCCCTTAA
	**ACTB-P**	ACCACCACCCAACACACAATAACAAACACA

Table S2 The demographic and clinical characteristics of patients in training, test and validation sets

	Training	Testing	Validation
Total (n)	132	45	91
Age - yr	55(29-83)	60.5(27-89)	58.5(24-86)
Gender – no.(%)			
Male	63	20	42
Female	69	25	49
Histology			
Normal	17	7	32
Hyperplastic Polys	5	1	0
LGIN	6	1	2
HGIN	0	0	22
Tumor			
Stage I	14	6	6
Stage II	34	5	5
Stage III	11	5	5
Stage IV	45	20	19

Table S3 Feature coefficient of the 4-marker model

TWIST1	C9orf50	KCNJ12	ZNF132
0.688	0.965	0.714	0.809
